# Sex differences in colonic gene expression and fecal microbiota composition in a mouse model of obesity-associated colorectal cancer

**DOI:** 10.1038/s41598-024-53861-z

**Published:** 2024-02-13

**Authors:** Yoo-Mee Chang, Yoo-Ree Kang, Yu-Gyeong Lee, Mi-Kyung Sung

**Affiliations:** Department of Food and Nutrition, College of Human Ecology, Sookmyung Women’s University, 100, Cheongpa-ro 47-gil, Yongsan-gu, Seoul, 04310 Republic of Korea

**Keywords:** Genetics, Microbiology, Systems biology, Biomarkers, Diseases, Gastroenterology

## Abstract

This study investigated the sex-specific correlation between obesity and colorectal cancer emphasizing a more pronounced association in males. Estrogen, chromosomal genes, and gut bacteria were assessed in C57BL6/J male, female and ovariectomized (OVX) female mice, subjected to either a low-fat diet (LFD) or high-fat diet (HFD) for 14 weeks. Induction of colon tumor involved azoxymethane (10 mg/kg) administration, followed by three cycles of dextran sulfate sodium. Male mice on HFD exhibited higher final body weight and increased colon tumors compared to females. Colonic mucin 2 expression was significantly higher in females. HFD-modulated differentially expressed genes numbered 290 for males, 64 for females, and 137 for OVX females. Only one up-regulated gene (*Gfra3*) overlapped between females and OVX females, while two down-regulated genes (*Thrsp* and *Gbp11*) overlapped between males and OVX females. Genes up-regulated by HFD in males were linked to cytokine-cytokine interaction, HIF-1 signaling pathway, central carbon metabolism in cancer. Sex-specific changes in gut microbial composition in response to HFD were observed. These findings suggest a male-specific vulnerability to HFD-induced colon tumor formation, implicating key genes and colonic bacteria in colon tumorigenesis.

## Introduction

Colorectal cancer, the third most common cancer in the world, is linked to several genetic and environmental factors^1,2^. Epidemiological studies have shown that the age-standardized incidence rate of colorectal cancer is higher in men than in women^3^. In addition, the correlation between colorectal cancer and obesity is more pronounced in men than in women^4–6^. Nevertheless, colorectal cancer is the second most common cancer in women aged > 65 years^3^ and older women have a relatively lower overall survival compared to age-matched men^7^, possibly owing to the loss of estrogen protection.^5^

Recently, the US National Institutes of Health has mandated the inclusion of sex as a biological variable while conducting biomedical research^8^. Biological sex plays an important role in the detection, prevention, and treatment of many diseases, including colorectal cancer^9^. In addition, fat distribution differs with sex, with women having less visceral and more subcutaneous adipose tissue than men^10^. As the accumulation of visceral adipose tissue increases the risk of colorectal cancer^11,12^, differences in incidence of colorectal cancer between men and women may be partly explained by differences in fat distribution. However, studies that reveal sex differences in the mechanism of colorectal cancer, particularly in relation to obesity, are lacking.

Sex differences in colorectal cancer have been mostly explained by differences in concentration of sex hormones. The Women Health Initiative study has revealed that hormone replacement therapy reduces the risk of colorectal cancer by 30% in postmenopausal women^13,14^. Estrogen treatment in mice with colitis-associated colorectal cancer has proven effective in reducing both the number and size of colon tumors^15^. The efficacy suggests that estrogen depletion plays a pivotal role in colorectal cancer development, particularly in postmenopausal women. Given that estrogen has the capacity to suppress excess fat accumulation, a factor positively correlated with the risk of colorectal cancer^16,17^, the rapid increase in colorectal cancer incidence in postmenopausal women may be attributed to estrogen depletion-induced fat accumulation. Recent insights propose that sex differences in cancer are genome-wide, involving genes located on both sex chromosome and autosomes^18^. Nevertheless, a comprehensive understanding of sex differences in the mechanism of obesity-associated colorectal cancer, particularly regarding chromosomal variances and estrogen-depletion-induced obesity, remains limited.

Chronic inflammation induced by obesity, alterations in adipokine concentration and gut dysbiosis have been suggested as causative factors in the occurrence of body fat-associated colorectal cancer^19^. The gut microbiota may influence the pathogenesis of obesity and related metabolic disorders, including colorectal cancer^20–22^. The gut microbiota can serve as a biomarker for early detection of colorectal cancer^23^. The proportion of *Hafnia alvei* belonging to the Proteobacteria phylum and that of *Akkermansia muciniphila* belonging to the Verrucomicrobia phylum increase in obesity and CRC patients^24^. In a recent study, AOM-treated *APC*^*Min/*+^ mice transplanted with feces from obese individuals exhibited increased colon tumor formation^25^. This observation implies that alterations in the gut microbial composition induced by obesity may impact the susceptibility to colorectal cancer. Sex differences exist in gut microbial composition^26^, and therefore, responses to high-fat diet (HFD) may vary by biological sex^27^. For example, the proportions of *Lactobacillus*, *Alistipes*, *Lachnospiraceae,* and *Clostridium* have been found to be increased by feeding HFD in the male group only^28^. In a separate investigation employing the AOM-DSS model for colorectal tumorigenesis, a reduction in microbial diversity and compositional changes were noted in female mouse compared to their male counterparts^29^.

This study aims to offer mechanistic insights into observed differences in the incidence of colorectal cancer among men, premenopausal women and postmenopausal women. We examined sex- and hormone-dependent variations by employing male (M), female (F) and ovariectomized (OVX)-F AOM-DSS colon tumorigenesis murine fed either LFD or HFD. Genes and gut bacteria composition associated with sex, diet and estrogen availability were identified. We utilized a high-fat diet-induced obesity model, widely acknowledged for its ability to closely emulate human obesity.

## Results

### Body and organ weights and number of tumors in experimental animals

**Figure 1:**
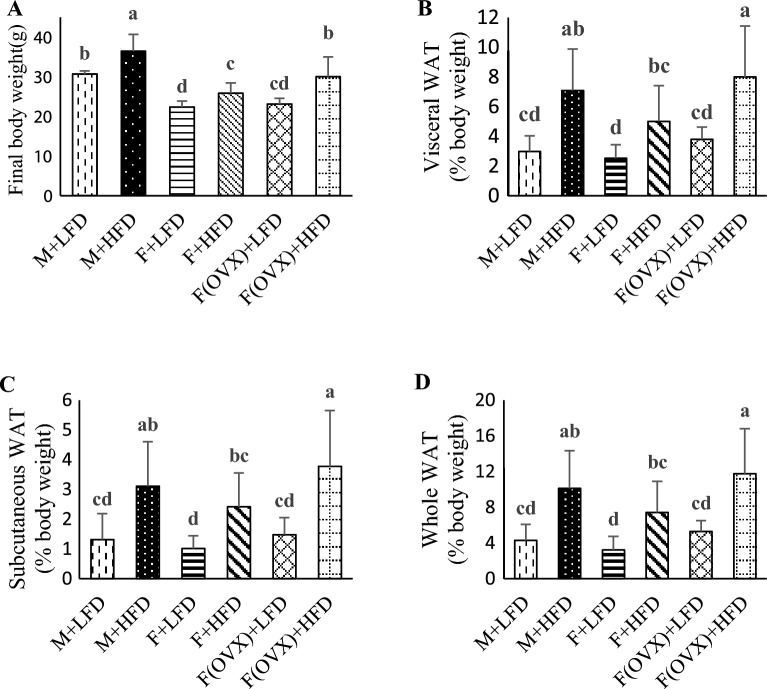
Final body weight and percentage of fat mass of experimental animals. C57BL/6J mice were fed LFD or HFD for 14 weeks. (**A**) Final body weight and the weights of adipose tissues including (**B**) visceral white adipose tissue (WAT), (**C**) subcutaneous WAT, and (**D**) whole WAT were measured at the end of the experiment. Results were analyzed using a one-way ANOVA and were expressed as mean ± standard deviation (SD). Bars labeled with different letters are significantly different based on Duncan's multiple range tests (*p* < 0.05).

**Figure 2:**
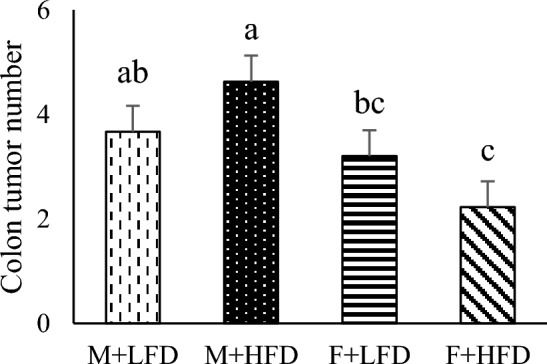
Effects of sex and diet and their interactions on the number of colon tumors. The number of colon tumors was measured at 75 days after AOM injection. Results were analyzed using two-way ANOVA and were expressed as mean ± standard deviation (SD). The p-values for main effects and their interactions are reported in box. Bars labeled with different letters are significantly different based on Duncan's multiple range tests (*p* < 0.05).

The final body weight (g) was significantly higher (p < 0.05) in the male M + HFD group (36.42 ± 4.29) than in the M + LFD group (30.64 ± 0.81) and all F groups (Fig. 1A). The final body weight (g) for the female groups F + LFD, F + HFD, F(OVX) + LFD, and F(OVX) + HFD was as follows: 22.32 ± 1.52, 25.82 ± 2.61, 23.10 ± 1.45, and 29.98 ± 5.02, respectively. Differences between LFD and HFD groups within female groups were statistically significant (p < 0.05). The average daily food intake for the groups M + LFD, M + HFD, F + LFD, F + HFD, F(OVX) + LFD and F(OVX) + HFD was as follows: 7.66 ± 0.26, 15.01 ± 3.63, 6.17 ± 0.20, 6.14 ± 0.17, 5.80 ± 0.07 and 6.73 ± 0.07, respectively. The experimental diet, which was freely accessible, contained a high saturated fat content, enhancing the diet’s palatability^30^. Consequently, we observed an increased average food intake in the M + HFD group (p < 0.05) compared to other groups. However, no statistically significant differences were observed within the female groups. These results may be partially explained by the observed hyperphagic response in male mice when fed a high-fat diet, which was not observed in male mice fed a low-fat diet or female mice fed either low-fat or high fat diet^31^. Consequently, body weight gain in the M + HFD mice can be attributed to higher energy intake. However, an increased body weight gain in females fed a HFD cannot be solely explained by hyperphagia. The weights of total white adipose tissue (g) for the groups M + LFD, M + HFD, F + LFD, F + HFD, F(OVX) + LFD and F(OVX) + HFD was as follows: 4.28 ± 1.79, 10.18 ± 4.22, 3.22 ± 1.52, 7.42 ± 3.47, 5.29 ± 1.21, and 11.77 ± 5.02, respectively. HFD-fed groups had significantly higher amount of white adipose tissue that their counterparts fed LFD (p < 0.05). (Fig. 1B–D). Estrogen deprivation through ovariectomy significantly reduced the uterine weights of OVX female mice. The liver weight was significantly higher in the M + HFD group than those in the other groups. The number of colon tumors in the M + HFD group was significantly higher than that in all female groups, and the F + HFD group had a significantly lower number of tumors than the M + LFD and M + HFD groups (Table 1). Two-way ANOVA showed a significant interaction between sex and diet (*p* < 0.05), indicating that HFD may be a risk factor in the male group only (Fig. 2). However, the effect of estrogen depletion on the number of colon tumors was not significant.

**Table 1:** Number of tumors in all experimental groups.

Group	Tumor number
M + LFD	3.67 ± 1.86^ab^
M + HFD	4.63 ± 1.19^a^
F + LFD	3.20 ± 1.14^bc^
F + HFD	2.22 ± 0.97^c^
F(OVX) + LFD	3.22 ± 1.20^bc^
F(OVX) + HFD	2.88 ± 1.46^bc^
p-value	< 0.05

Results were analyzed using one-way ANOVA and are expressed as mean ± standard deviation (SD). Means with different superscripts are significantly different based on Duncan's multiple range tests (*p* < 0.05).

### Colonic MUC2 expression

**Figure 3:**
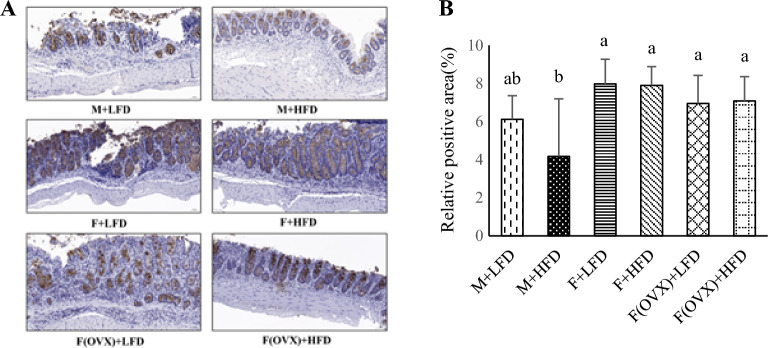
Immunohistochemical analysis of MUC2 expression. (**A**) Representative images of MUC2 expression in the colon (20 × magnification) and (**B**) percentage of MUC2 staining area. MUC2 staining is visualized by a brown color. Results were analyzed using one-way ANOVA and are expressed as mean ± standard deviation (SD). Bars labeled with different letters are significantly different based on Duncan's multiple range tests (*p* < 0.05).

Colonic MUC2 expression as a protective factor against epithelial damage was measured as an indicator of the integrity of mucosal barrier. Immunohistochemical staining indicated that MUC2 expression (%) for the groups M + LFD, M + HFD, F + LFD, F + HFD, F(OVX) + LFD and F(OVX) + HFD was as follows: 6.12 ± 1.25, 4.17 ± 3.03, 7.98 ± 1.30, 7.91 ± 0.98, 6.96 ± 1.47 and 7.09 ± 1.28. (Fig. 3A,B). Results indicated that the percentage of MUC2 positive area is significantly lower in the M + HFD group than in the female groups (p < 0.05). No significant difference was observed between the M + LFD and M + HFD groups.

### Sex differences in gene expression of HFD-modulated colon tissues

**Figure 4:**
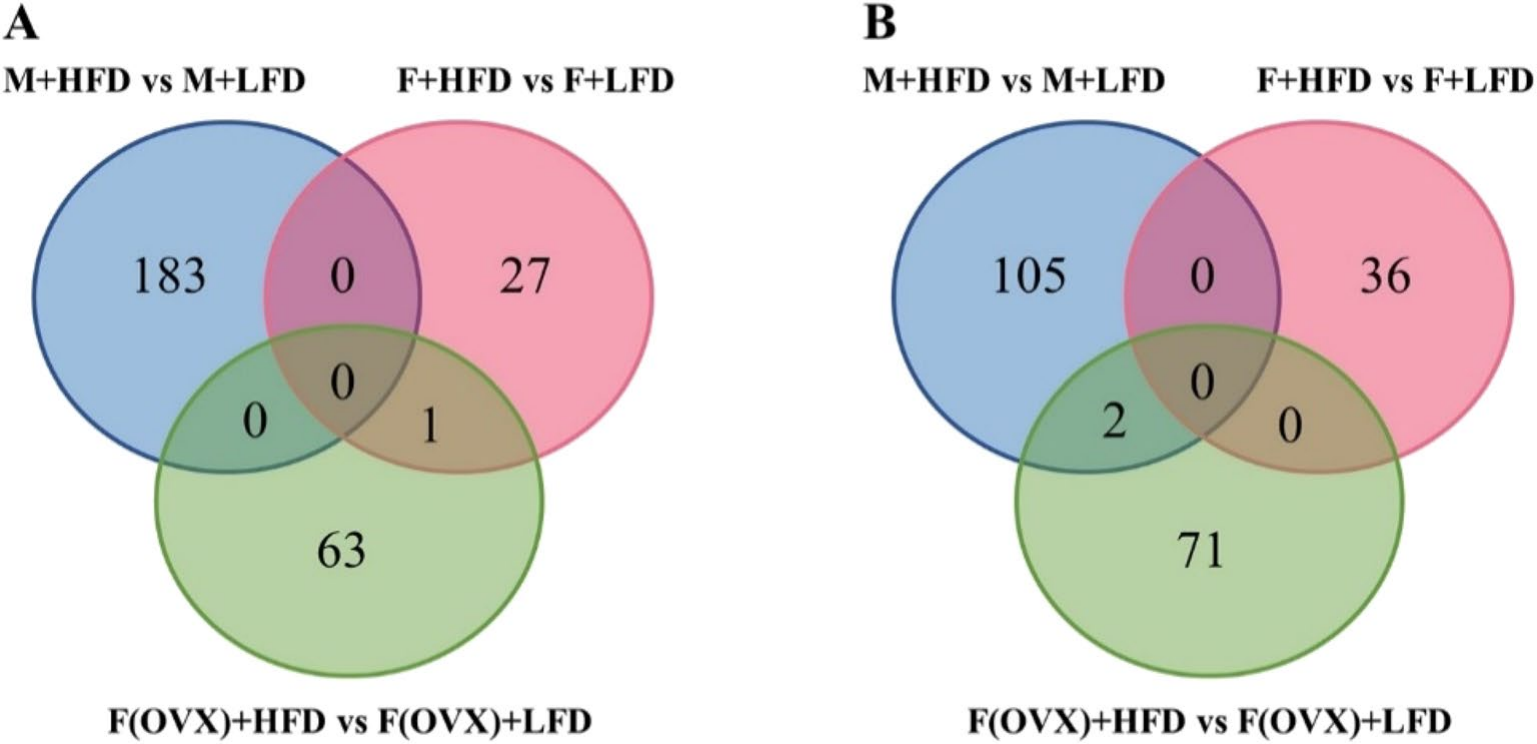
Venn diagram comparing (**A**) upregulated DEGs and (**B**) downregulated DEGs in colon between LFD- and HFD-fed groups in male, female, and OVX mice. Fold change values > 1.5 were considered upregulated whereas those < -1.5 were downregulated along with p-value threshold of 0.05 for statistically significant differences.

Microarray analysis of colon tissues was performed to determine differentially expressed genes (DEGs) in HFD-fed vs. LFD-fed groups in males, females or OVX females. The DEGs obtained from three pairwise comparisons were plotted in a Venn diagram (Fig. 4A,B) revealing that the number of HFD-modulated DEGs was 290 for males, 64 for females, and 137 for OVX females. Only one up-regulated gene (Gfra3) overlapped between females and OVX females, whereas 2 down-regulated genes (Thrsp and Gbp11) overlapped between males and OVX females. Therefore, HFD affected more genes in male mice than in female mice, which might be associated with tumor development.

### Kyoto encyclopedia of Genes and Genome (KEGG) enrichment analysis of male-specific upregulated or downregulated DEGs

**Figure 5:**
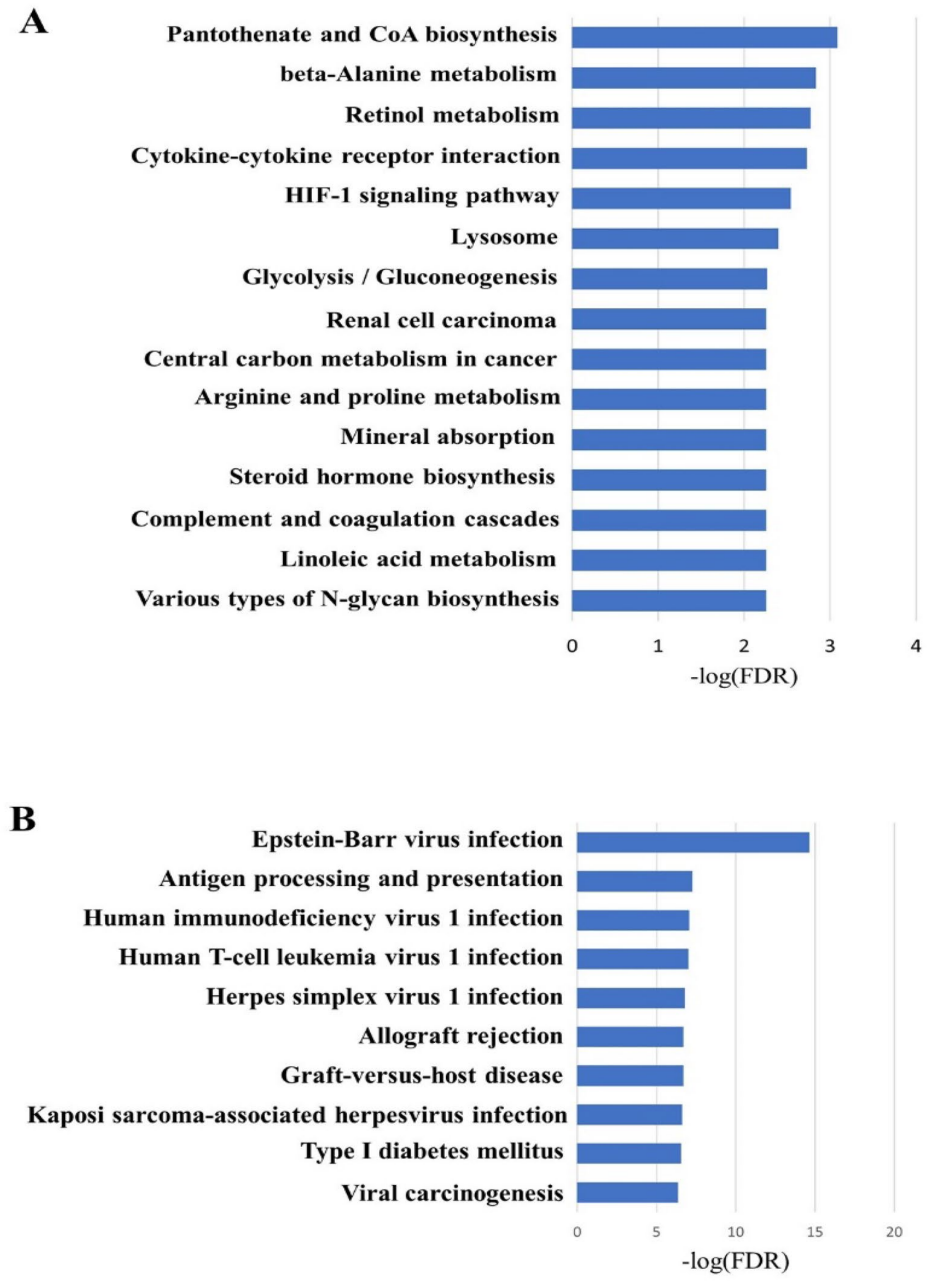
(**A**) Top 15 enriched KEGG pathways of male-specific upregulated DEGs. (**B**) Top 10 enriched KEGG pathways of male-specific downregulated DEGs.

A total of 288 male-specific DEGs, including 183 upregulated and 105 downregulated genes, were subjected to KEGG analysis to identify the major pathways these genes are involved in. We aimed to identify genes and their functions that may accelerate tumor formation in the M + HFD group. The top 15 significant KEGG pathways related to male-specific upregulated DEGs included several cancer-related pathways, such as interaction between cytokines and cytokine receptors, hypoxia-inducible factor (HIF)-1 signaling pathway, and central carbon metabolism in cancer (Fig. 5A). Male-specific downregulated DEGs were mainly involved in viral infection-related signaling pathways such as infection by Epstein-Barr virus, herpes simplex virus 1, human T-cell leukemia virus 1, and human immunodeficiency virus 1 (Fig. 5B).

### Hub gene identification in male-specific DEGs using protein–protein interaction (PPI) network construction

**Figure 6:**
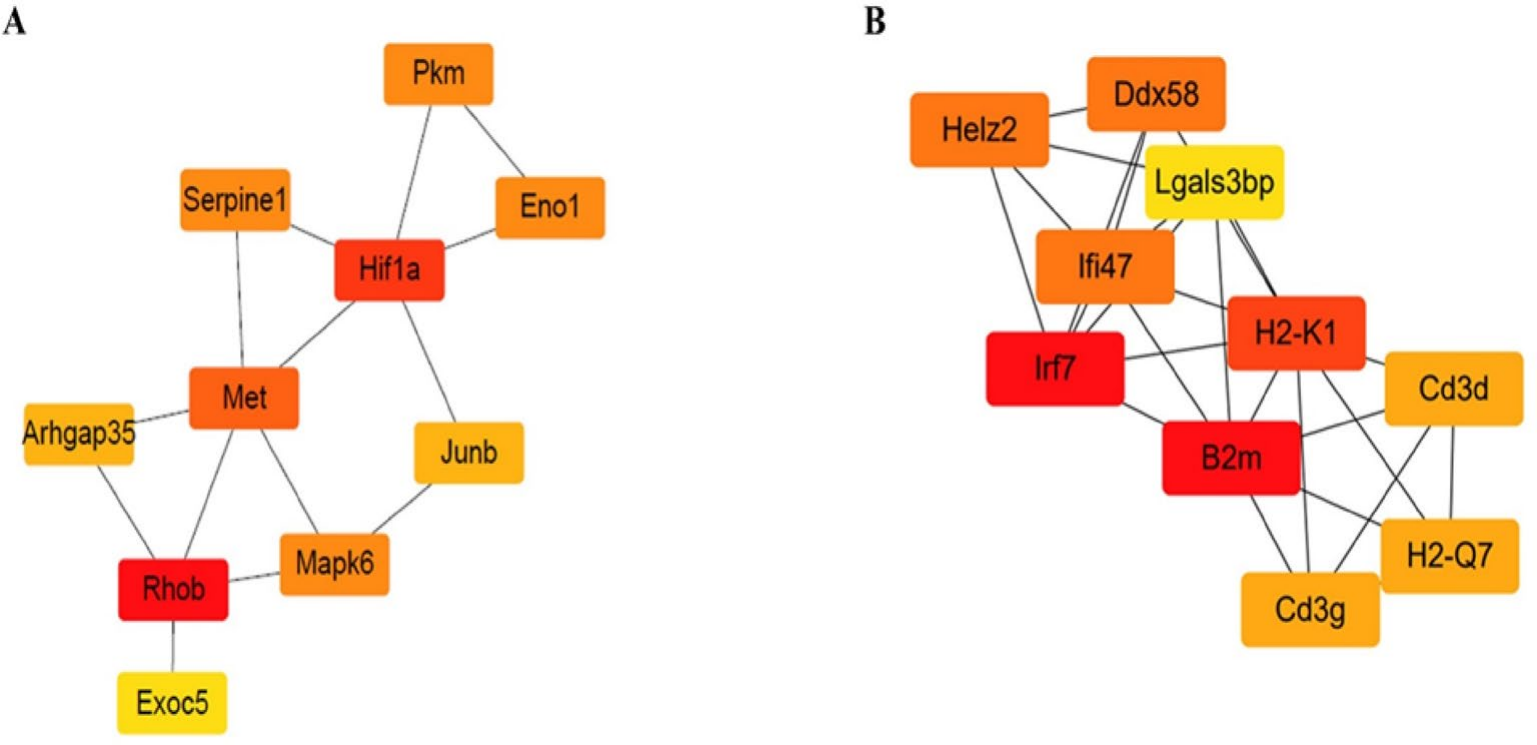
Top 10 hub genes among the (**A**) upregulated and (**B**) downregulated DEGs in male mice. The top 10 hub genes were screened by cytoHubba according to degree. Red color represents the highest degree, and yellow color represents the lowest degree.

PPI networks of the male-specific upregulated or downregulated DEGs were constructed based on the information obtained from the STRING database. The PPI network of upregulated DEGs included 139 nodes and 97 edges. Among them, 10 genes (*Rhob, Hif1a, Met, Pkm, Eno1, Mapk6, Serpine1, Arhgap35, Junb*, and *Exoc5*) with the highest degree scores were identified as male-specific upregulated hub genes by applying the cytoHubba plugin (Fig. 6A). The PPI network of downregulated DEGs included 86 nodes and 87 edges. The top 10 male-specific downregulated hub genes with higher degrees were filtered, including *Irf7, B2m, H2-K1, Helz2, Ddx58, Ifi47, Cd3d, H2-Q7, Cd3g*, and *Lgals3bp* (Fig. 6B).

### Sex-specific changes in the composition of gut microbiota in response to HFD

**Figure 7:**
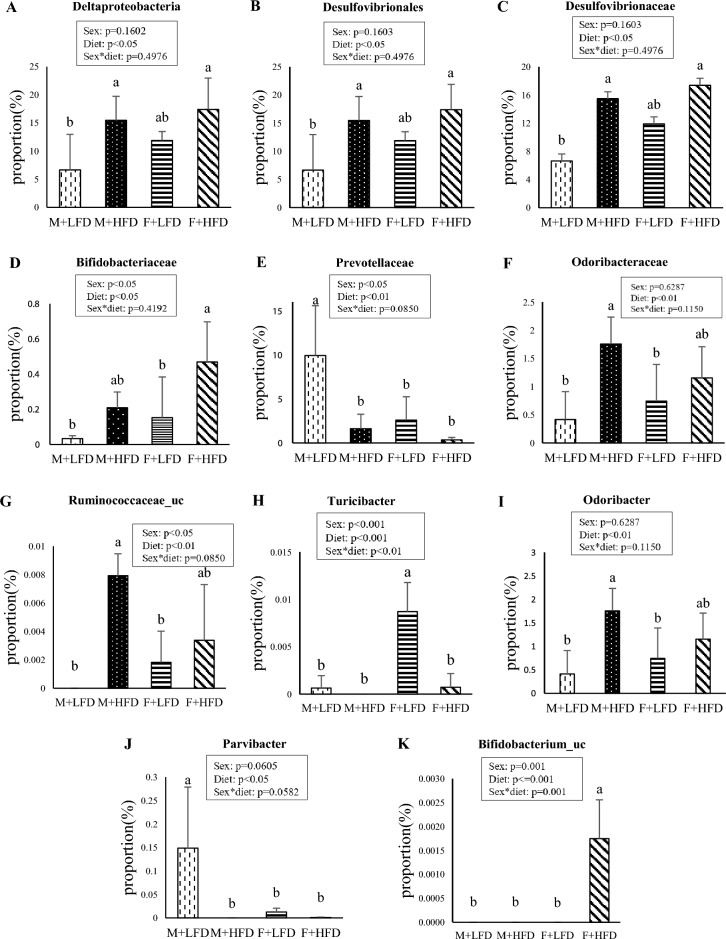
Effects of sex and diet and their interactions on gut microbial composition at (**A**) class, (**B**) order, (**C**–**F**) family, (**G**–**J**) genus, and (**K**) species levels. Results were analyzed using two-way ANOVA and are expressed as mean ± standard deviation (SD). The p-values for main effects owing to sex and diet and their interaction are reported in box. Bars labeled with different letters are significantly different based on Duncan's multiple range tests (*p* < 0.05).

Effects of sex and diet, and their interactions on the composition of gut microbiota were determined by two-way ANOVA at each level of microbial taxonomy (Fig. 7). At the class level, diet had a significant effect on the Deltaproteobacteria [F(1,12) = 8.97, p = 0.011]. At the order level, diet had a significant effect on the relative abundance of Desulfovibrionales [F(1,12) = 8.97, p = 0.011]. At the family level, diet had a significant effect on the relative abundance of Desulfovibrionaceae [F(1,12) = 8.97, p = 0.011] and Odoribacteraceae [F(1,12) = 10.20, p = 0.007]. There were significant effects of both sexes [F(1,12) = 7.07, p = 0.021] and diet [F(1,12) = 10.61, p = 0.007] on the relative abundance of the family Prevotellaceae. We also observed significant effects of both sex [F(1,12) = 5.04, p = 0.044] and diet [F(1,12) = 8.52, p = 0.013] on the relative abundance of the family Bifidobacteriaceae. The proportion of Deltaproteobacteria (class level), Desulfovibrionales (order level), and Desulfovibrionaceae (family level), corresponding to sulfate-reducing bacteria, significantly increased in the M + HFD group compared to that in the M + LFD group. At the genus level, taxa with significant interaction effects between sex and diet included *Ruminococcaceae_uc* [F(1,12) = 7.20, p = 0.012] and *Turicibacter* [F(1,12) = 16.34, p = 0.002]. Diet had a significant effect on the relative abundance of bacterial genera, including *Odoribacter* [F(1,12) = 10.20, p = 0.008] and *Parvibacter* [F(1,12) = 6.02, p = 0.030]*.* In the male group, the ratio of *Odoribacter* was significantly increased by HFD, and the ratio of *Parvibacter* was significantly decreased. At the species level, a significant effect of interaction of sex and diet was noticed on the relative abundance of *Bifidobacterium_uc* [F(1,12) = 18.65, p = 0.001].

### Correlation of bacteria at family, genus, species levels with the number of tumors and weight of adipose tissue

*Ruminococcaceae_uc* (genus level), the abundance of which was significantly increased by HFD in the male group, showed a positive correlation with the number of colon tumors (R = 0.55) (Table 2). The abundance of *Turicibacter* (genus level) was significantly higher in the F + LFD group than in all other groups, and relative abundance of *Turicibacter* showed a negative correlation with the weight of whole white adipose tissue (R = − 0.48). Odoribacteraceae (family level) and *Odoribacter* (genus level), which were significantly increased by HFD in the male group, showed a positive correlation with the weight of whole white adipose tissue (R = 0.42).

**Table 2:** Correlation of bacteria with the number of tumors and weight of adipose tissue.

Taxa		Number of tumors* (R value)	Weight of adipose tissue* (R value)
Family	Odoribacteraceae		0.4244
Genus	Ruminococcaceae_uc	0.5519	
	Odoribacter		0.4244
	PAC000198_g		− 0.5676
	PAC001512_g		− 0.4197
	PAC002390_g		0.4962
	Turicibacter		− 0.4773
Species	PAC000701_s		
	PAC001060_s		
	EU791023_s		− 0.4189
	PAC001075_s		− 0.5680
	PAC001078_s		− 0.4803
	PAC002390_s		0.4962
	PAC002410_s		
	PAC001637_g_uc		0.4381
	PAC001713_s		0.5552
	Parabacteroides_uc		− 0.4451

*Pearson correlation coefficients (r) were obtained to determine the relationships between bacteria and the number of tumors or weights of adipose tissue using Pearson's correlation analysis. Significance was set at *p* < 0.05.

### Functional differences in the gut microbiota between male and female mice

**Figure 8:**
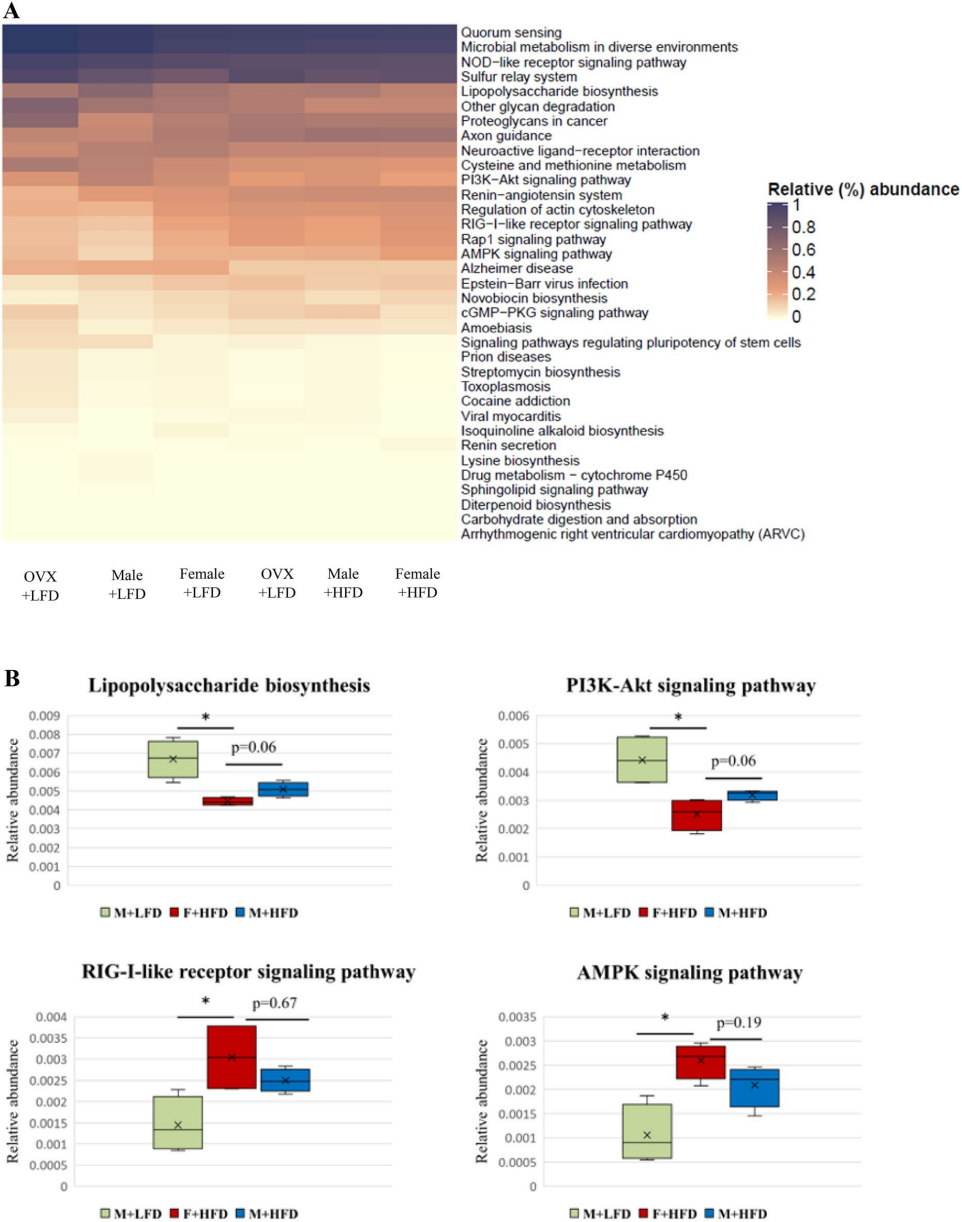
(**A**) Heatmap of metabolic pathways of all experimental groups obtained from PICRUSt analysis of 16S rRNA gene sequence data. Pathways are colored based on their abundance relative to that of all measured genes. (**B**) Boxplots showing the significantly different KEGG pathways between male and female mice.

The potential functions of gut microbiota that showed compositional differences were determined using the PICRUSt and MinPath algorithms and are presented in Fig. 8A. Microbial gene functions, lipopolysaccharide biosynthesis, and phosphoinositide-3-kinase–Akt signaling pathway were significantly higher in the M + LFD group than in the F + HFD group (Fig. 8B). Microbial gene functions, AMP-activated protein kinase signaling pathway, and retinoic acid-inducible gene I-like receptor signaling pathway were significantly lower in the M + LFD group than in the F + HFD group (Fig. 8B).

## Discussion

In this study, the number of colon tumors in the M + HFD group was significantly higher than that in all female groups. The effect of sex on the number of colon tumors and the effect of interaction between sex and diet were significant. This may support the findings of previous epidemiological studies showing that the correlation between obesity and colorectal cancer was stronger in men than in women^32,33^. We also found that the expression of MUC2 that forms the protective mucus layer in the large intestine was significantly higher in all female groups than in the M + HFD group. *MUC2* knockout mice have been previously found to be more susceptible to colon tumorigenesis, and MUC2 deficiency resulted in increased cell proliferation, reduced apoptosis, and elevated c-Myc levels in tumors^34^. The downregulation of MUC2 increases the levels of Ki67 and matrix metalloprotease-9, suggesting an association between MUC2 and cellular proliferation and invasion^35^. Induction of murine colorectal cancer through oncogenic K-Ras expression results in a significant reduction of MUC2 expression in HFD group compared to that in the normal diet-fed group^36^. In Winnie mice, *MUC2* mutations cause endoplasmic reticulum stress in intestinal goblet cells, resulting in depletion of the mucus barrier and increased bacterial translocation leading to spontaneous colitis^37^. Taken together, this study reveals that diet- and sex-specific changes in MUC2 expression may be associated with an increased risk of colorectal cancer.

In this study, male-specific upregulated DEGs were mainly involved in cancer-related pathways, such as cytokine–cytokine receptor interaction, HIF-1 signaling, and central carbon metabolism. Indeed, obesity-induced chronic inflammation is a major link between obesity and colorectal cancer^19^. Inflammatory cytokines such as interleukin (IL)-6, tumor necrosis factor alpha, and CC motif chemokine ligand 2 produced in adipose tissue are major factors that induce adiposity-related chronic inflammation^38,39^. Specifically, IL-6 is overexpressed in colorectal cancer tissues, and an increase in serum IL-6 concentration has been found to correlate with an increase in tumor size and reduced survival rate^40^. HIF-1 is a key factor that regulates cellular adaptation to hypoxia, and elevated HIF-1 levels are strongly correlated with tumor metastasis, angiogenesis, and poor patient prognosis^41^. To determine the major genes that play a role in HFD-linked colorectal cancer development, male-specific upregulated hub genes were selected using the degree algorithm of the cytoHubba plug-in. The top 10 hub genes were *Rhob, Hif1a, Met, Pkm, Eno1, Mapk6, Serpine1, Arhgap35, Junb*, and *Exoc5*. The expression of RhoB is increased by stress-induced signaling events, such as lipopolysaccharides, inflammatory cytokines, and growth factors^42^. An increased RhoB expression contributes to the development of ulcerative colitis by regulating cell signals and inducing changes in intestinal microbial composition and metabolites^43^. *Met* expression is significantly increased in the early stages of colorectal cancer^44^, and *MAPK6* promotes cancer development by activating Akt in an mTORC2-independent manner^45^. Pyruvate kinase isoenzyme type M2 (PKM2) encoded by *PKM* increases anabolic synthesis of macromolecules through the pentose phosphate pathway and consequently promotes proliferation and growth of cancer cells^46,47^. Taken together, *RhoB, HIF1A, Met, MAPK6*, and *PKM* may play key roles in promoting colorectal tumor formation in males.

In this study, we found that changes in the composition of gut microbes caused by HFD differed according to sex. In the male group, the proportions of *Odoribacter* (genus level) and *Ruminococcaceae_uc* (genus level) were significantly increased, and the abundance of *Parvibacter* (genus level) was significantly decreased by HFD. In the female group, however, the proportions of Bifidobacteriaceae (family level) and *Bifidobacterium_uc* (species level) were significantly increased, and the abundance of *Turicibacter* (genus level) was significantly decreased by HFD. The proportion of *Odoribacter* has been found to be higher in AOM/DSS-treated mouse model of cancer than in control mice^48^, and patients with colorectal cancer show the same pattern^49^. We observed a positive correlation between *Odoribacter* and the weight of whole white adipose tissue, suggesting that *Odoribacter* may play a role in tumor formation in males. Ruminococcaceae is increased in patients with colorectal cancer compared to that in healthy people^50^. Moreover, the proportion of Ruminococcaceae is increased in HFD-fed mice compared to that in mice fed a chow diet^51^. Since we observed a positive correlation between *Ruminococcaceae_uc* and the number of colon tumors, *Ruminococcaceae_uc* seems to be closely related to tumor formation in males. *Parvibacter* improves the function of gut barrier, increases the expression of ZO-1 corresponding to the tight junction protein, and reduces the entry of LPS into the body^52^. The proportion of *Parvibacter* is negatively correlated with body weight and weight of white adipose tissue^53^. In our study, the abundance of *Parvibacter* decreased significantly in the M + HFD group compared with that in the M + LFD group. This indicates that a decrease in *Parvibacter* ratio may also have contributed to tumor development in males. In addition, we found that the abundance of sulfate-reducing bacteria, including Deltaproteobacteria (class level), Desulfovibrionales (order level), and Desulfovibrionaceae (family level), was significantly increased by HFD in the male group. Hydrogen sulfide produced by sulfate-reducing bacteria can promote cancer development by inducing oxidation and DNA damage^54^. In mice with aberrant colonic crypt induced by AOM, significant increases in Deltaproteobacteria and Desulfovibrionales have been observed in the HFD group compared to that in the control diet group^55^. Taken together, sulfate-reducing bacteria may also play a role in tumor development.

*Bifidobacterium_uc* that belongs to the family Bifidobacteriaceae was significantly more abundant in the F + HFD group than in all other groups, and *Turicibacter* was significantly more abundant in the F + LFD group than in all other groups. In mice, *Bifidobacterium* and *Turicibacter* have been reported to be more abundant in females than in males^48^. Bifidobacteriaceae increases the production of short-chain fatty acids and strengthens the function of gut barrier^56^. *Turicibacter* has been reported to have a positive correlation with the butyric acid production^57^. Moreover, the abundance of *Turicibacter* has been found to be decreased in animal models of inflammatory bowel disease^58,59^ and in HFD-induced obese mice^60^. In our study, *Turicibacter* was negatively correlated with the weight of whole white adipose tissue. Therefore, *Turicibacter* may have a diet-dependent effect on sex differences in colorectal cancer. In addition, our results showed the F + HFD group possessed less microbiota involved in tumor-promoting pathways and more microbiota involved in tumor-suppressing pathways than did the M + LFD group, suggesting the possible involvement of gut microbiota in colon carcinogenesis.

The strength of this study lies in its capacity to provide indispensable data essential for tailored colorectal cancer prevention and treatment, extending beyond the consideration of estrogen levels and incorporating a genomic perspective. It aims to elucidate the mechanisms contributing to the higher incidence of colorectal cancer in men and the possible increase in postmenopausal women compared to their premenopausal counterparts. A limitation, however, is that the selection of genes and bacteria is based on group-to-group comparisons. To identify actionable targets, it would be necessary to delve further into functional studies to establish causality.

In summary, high-fat diet accelerates colorectal tumor formation in male mice not in both ovary intact or ovariectomized female mice suggesting estrogen depletion might not be solely responsible for increased tumor formation. The number of HFD-modulated group-specific DEGs was 290 for males, 64 for females, and 137 for OVX females. Genes up-regulated HFD males include pathways related to cytokine-cytokine interaction, HIF-1 signaling pathway, central carbon metabolism in cancer, all of which are positively associated with cancer development. Sex-specific alterations in gut microbial composition in response to HFD in males involve elevated proportions of *Odoribacter* and *Ruminococcaceae_uc*, accompanied by a reduction in *Parvibacter*.

## Methods

### Animals and experimental design

**Figure 9:**
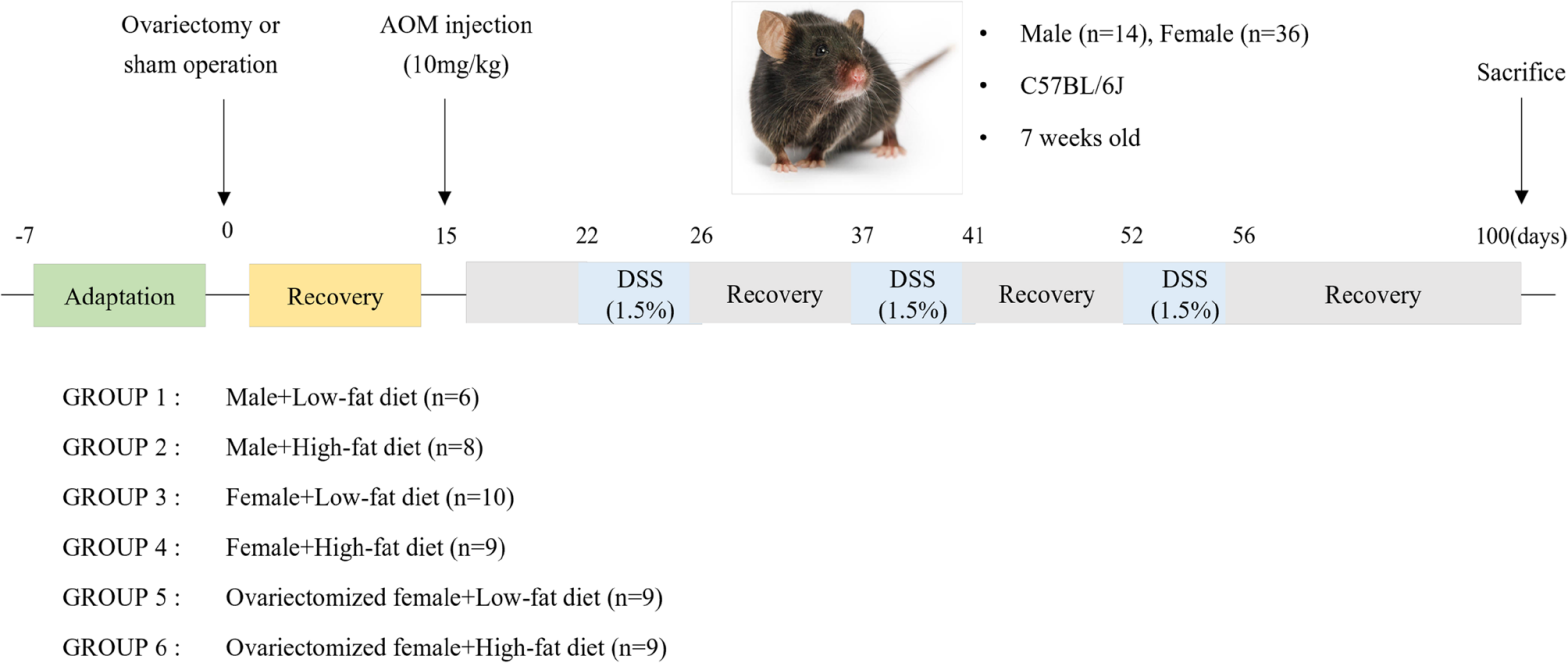
Experimental design of the study.

In this study, we employed a high-fat diet-induced obesity model to explore the influence of sex hormones and genes as factors contributing to sex-specific differences in obesity-related colorectal cancer. We also investigated their associations with the composition of gut bacteria. This choice of model was made in preference to commonly used genetically modified obesity models, such leptin-deleted ob/ob, leptin receptor-deleted db/db or adiponectin-deleted model. The decision was based on previous reports suggesting that leptin and its receptor, as well as adiponectin, possess signaling capabilities that promote the development of colorectal tumors^61–63^. Simultaneously, we considered the composition of gut microbiota, which can be significantly altered by dietary factors. This parameter served as another indicator of the mechanisms at play, further justifying the used of the diet-induced obesity model as a more human-relevant approach. Seven-week-old male and female C57BL/6J mice purchased from Charles River Laboratories (Yokohama, Japan) were housed in plastic cages (2 ~ 3 mice/cage) under controlled temperature (21 ± 2 °C), humidity (50 ± 10%) and light (12 h light/dark cycle) conditions. After one-week acclimatization, sham surgery was performed on male mice (n = 14), and ovariectomy (n = 17) or sham surgery (n = 19) was performed on female mice. After 14 days of recovery period, male, female or ovariectomized female animals were allocated into two dietary groups: a low-fat diet (LFD; 10 kcal% fat) group or a HFD (45 kcal% fat) diet group (Fig. 9). The experimental groups included M + LFD (n = 6), M + HFD (n = 8), F + LFD (n = 10), F + HFD (n = 9), OVX + LFD (n = 9), and OVX + HFD (n = 8). After sorting by body weight, mice were alternately allocated into two groups in descending order to ensure similar baseline average body weight in both LFD group and HFD group. Colitis-associated colorectal cancer was induced by a single intraperitoneal injection of 10 mg/kg azoxymethane (AOM) at day 15, followed by 1.5% (w/v) DSS in drinking water starting one week after AOM injection. Mice received three cycles of DSS treatment, each consisting of 5 consecutive DSS supply at 10-day intervals. A healthy control group was not included in this study, as the primary objective was to investigate hormonal and genetic determinants in colitis-associated CRC. The experimental diet was supplied for 14 weeks and body weight and food intake were measured three times a week. At 75 days after AOM injection, mice were sacrificed under anesthesia using a 2:1 mixture of Zoletil (Virbac, Magny-en-Vexin, France) and Rompun (Bayer, Seoul, Republic of Korea). We used the “weight loss exceeding 20% of the initial weight” as the humane end point. Colon tissues from the anus to below the cecum were excised using scissors and opened longitudinally along the main axis^64^.Feces were washed with phosphate-buffered saline, and the colons were laid flat on a glass plate. The total number of tumors (nodular, polypoid, or caterpillar-like) in the entire colon was counted^65^. After macroscopic evaluation of tumors, approximately 1 cm of the distal colon (1.5 cm proximal to the anus) was fixed with formalin at room temperature for histological analysis (n = 4). We selected the four animals with the closest average body weight for each group. Rest of the distal colon was immersed in RNA later (Thermo Fisher, Waltham, MA, USA) solution for microarray analysis (n = 5) and stored at -80 °C. Fecal samples were collected from all the animals in each group three days before sacrifice and stored at − 80 °C until further analysis. The sample size was determined through power analysis based on the number of colon tumor, which served as a primary outcome. This study was approved by the Institutional Animal Care and Use Committee of the Sookmyung Women’s University, Seoul, Republic of Korea (SMWU-IACUC-2012–015-02) and followed the Korean Institutional Animal Care and Use Committee (IACUC) guidelines (Korea Food & Drug Administration, Chungju, Republic of Korea). This study is reported in accordance with Animal Research: Reporting of In Vivo Experiments (ARRIVE) guidelines.

### Immunohistochemical analysis

Immunohistochemical staining was performed on colon tissue samples (n = 4 per group). We selected four animals with the closest average body weight for each group. Colon tissues were fixed in 10% buffered formalin and embedded in paraffin. Paraffin-embedded sections (5 µm) were analyzed using rabbit anti-MUC2 antibody (Abcam, Cambridge, MA, USA). Slides were examined using an Olympus BX51 microscope (Olympus, Tokyo, Japan) at 20–400 × magnification. Quantitative analyses were performed using the Image-Pro 10 program, and the ratio of positively stained area to the total area was calculated for each sample. Three random scans per section were analyzed and averaged.

### Microarray analysis and identification of DEGs

Colon tissue microarray analysis was conducted using five animals from each group, selected based on the closest average body weight. Total RNA was extracted from colon tissues using TRIzol reagent (Thermo Fisher Scientific, Waltham, MA, USA). RNA purity was assessed from the ratio of absorbances at 260 and 280 nm using a ND-2000 NanoDrop (Thermo Fisher Scientific, USA), with values between 1.7 and 2 considered acceptable. Total RNA integrity was checked using an Agilent Technologies 2100 Bioanalyzer (Agilent Technologies, Santa Clara, CA, USA), with an RNA Integrity Number value ≥ 7. Raw data were extracted using the Agilent Feature Extraction Software (v11.0.1.1) and were summarized for the same gene automatically using the Agilent feature extraction protocol to generate a raw data text file, providing expression data for each gene probed on the array. Array probes with Flag A in samples were filtered out. The selected gProcessedSignal value was logarithmically transformed and normalized using the quantile method. Statistical significance of the expression data was determined using fold change and independent *t*-test, in which the null hypothesis was that no difference existed among groups. Fold change ≥ 1.5 and *p*-value < 0.05 were regarded as the cut-off criteria to determine DEGs. Three comparison groups of DEGs were generated: M + LFD vs. M + HFD, F + LFD vs. F + HFD, and F(OVX) + LFD vs. F(OVX) + HFD. We generated a Venn diagram that showed the relationships among DEGs in these three pairwise comparison groups.

### Functional enrichment analysis of DEGs

The KEGG database contains network-related information on genes and molecules^66^. In our study, KEGG enrichment analysis was performed using the g:Profiler tool (https://biit.cs.ut.ee/gprofiler/) to map DEGs to specific biochemical pathways in the KEGG database^67^. The statistical significance of KEGG pathways was determined using genes with false discovery rate (FDR) < 0.05, and the enrichment score [− log10 (FDR)] was calculated. The top ten KEGG pathways were sorted in descending order based on the enrichment score. When multiple pathways had the same enrichment score, more than 10 pathways were selected.

### Construction of PPI network and hub gene identification

To confirm the interactions among DEGs, a PPI network was constructed using the STRING database (https://string-db.org). The criterion for PPI network included a confidence score ≥ 0.4. In a PPI network, each gene is represented as a node, and the interactions between genes are represented by edges^68^. Degree represents the number of edges connected to a given node, and nodes with a high degree are commonly referred to as hub genes^69^. In our study, hub genes were selected in the order of high-degree scores using the degree algorithm of cytoHubba that identifies hub objects and subnetworks in a complex interactome^70^.

### Analysis of the gut microbiota

Fecal bacterial DNA was extracted using a QIAamp DNA Stool Mini Kit (Qiagen, Hilden, Germany). Amplification by polymerase chain reaction (PCR) was performed using primers targeting the V3–V4 region of the 16S rRNA gene. Thermal cycling consisted of an initial denaturation at 95 °C for 3 min, followed by 25 cycles of denaturation at 95 °C for 30 s, annealing at 55 °C for 30 s, and extension at 72 °C for 30 s, and a final elongation at 72 °C for 5 min. Non-target products were removed using CleanPCR (CleanNA), and the quality and size of PCR products were confirmed using a Bioanalyzer 2100 (Agilent, Palo Alto, CA, USA). Mixed amplicons were pooled and sequenced using an Illumina MiSeq Sequencing system (Illumina, San Diego, CA, USA). Data analysis was performed using the EzBioCloud database (ChunLab Inc., Seoul, Republic of Korea). Based on 16 s rRNA gene sequences, differences in the composition of gut microbiota between experimental groups were verified, and the potential function of gut microbiota was predicted using the PICRUSt and MinPath algorithms. The basic functional profiles were estimated using PICRUSt, and MinPath was used to predict the metabolic pathways. MinPath is used to minimize the number of pathways required to map all functions by handling the problem of pathway redundancy. KEGG pathways that were differentially expressed in all experimental groups were identified and visualized as a heatmap.

### Statistical analysis

Statistical analysis was performed using SAS 9.4 program (SAS Institute Inc., Cary, NC, USA), and all values are presented as mean ± standard deviation (SD). Data were analyzed using one-way analysis of variance (ANOVA) or two-way ANOVA followed by Duncan's multiple range test. *p* < 0.05 was considered statistically significant. Two-way ANOVA was performed on the four groups (M + LFD, M + HFD, F + LFD, and F + HFD) to determine the main effects of sex and diet separately and their interactions. The Mann–Whitney U-test was used to compare differences between two groups without normal distributions, and the Kruskal–Wallis H test (non-parametric ANOVA) was used to compare differences between more than two groups. Correlations between the relative abundance of gut bacteria and the number of tumors or weight of adipose tissue were evaluated using the Pearson’s correlation test.

### Ethics approval

The protocols involving mice were approved by the Institutional Animal Care and Use Committee of Sookmyung Women`s University, Seoul, Republic of Korea (SMWU-IACUC-2012-015-02).

## Data Availability

The datasets generated by microarray experiments and analyzed during the current study are available in the Gene Expression Omnibus database GEO GSE239516. Other raw data sets generated during the current study are available from the corresponding author on reasonable request.
